# The synergic inhibitory effects of dark tea (Camellia sinensis) extract and p38 inhibition on the growth of pancreatic cancer cells

**DOI:** 10.7150/jca.34637

**Published:** 2019-10-21

**Authors:** Ke Zheng, Qin Zhao, Qing Chen, Weiqun Xiao, Yuedeng Jiang, Yuhui Jiang

**Affiliations:** 1 The Institute of Cell Metabolism and Disease, Shanghai Key Laboratory of Pancreatic diseases, Shanghai General Hospital, School of Medicine, Shanghai Jiaotong University, Shanghai, 200080, P.R.China.; 2 The office of Anhua Tea Industry and Tea Culture Development Leading Group, Hunan, 413500, P.R.China.

**Keywords:** Dark tea, water eluate, pancreatic cancer, p38 inhibition, ID1.

## Abstract

**Background:** Dark tea is one of the most popular types of Chinese tea, which has been reported to exhibit anti-obesity, anti-oxidation and antitumor activities in according human cell lines. In terms of tumorigenesis, the systemic study of the physiological effect of specific fraction of dark tea and the relevant molecular mechanism warrant more attention.

**Methods:** Dark tea was firstly isolated through solvent extraction method. Dissolved ethyl acetate extract was further fractioned by elution with various concentration of ethyl alcohol. The cytotoxicity effect of dark tea on cell proliferation was evaluated by CCK8 assay in HPDE human normal pancreatic duct epithelial cells, SW1990 and PANC-1 human pancreatic cancer cells, and SW1116 human colorectal cancer cells. Immunoblotting and flow cytometry analysis were utilized to examine the status of protein and reactive oxygen species respectively. Gene expression profile was analyzed by cDNA microarray and real-time PCR. The plasmid for ID1 expression was stably transfected into SW1990 cells for relevant functional analysis. The effect of dark tea extract on tumorigenesis *in vivo* was studied in xenograft tumor model.

**Results:** Water eluate fraction of the ethyl acetate extract from dark tea inhibited the growth of SW1990, PANC-1 and SW1116 cells more efficiently compared with that in HPDE cells. Meanwhile, p38 activity was increased and AKT activity was dropped in cancer cells with dark tea extract treatment. Further functional analyses indicated that water eluate fraction and p38 inhibitor treatment exerted a synergic inhibitory effect on cancer cells growth, which was related to their suppressive effect on expression level of ID1 (inhibitor of differentiation protein 1), which was highly expressed in cancer cells. The analysis utilizing xenograft tumor model further indicated water eluate fraction exhibited a significantly inhibitory effect on tumorigenesis.

**Conclusion:** Based on the sequential extraction procedure, our results reveal the inhibitory effect of water eluate fraction of the ethyl acetate extract from dark tea and its synergistic effect with p38 inhibition on the growth of pancreatic cancer cells, in which ID1 is identified as a downstream effector. This sheds insights into the physiological relevance of specific fraction of dark tea to tumorigenesis in pancreatic cancer.

## Introduction

Pancreatic cancer is the seventh leading cause of cancer death worldwide 1, which is typically diagnosed at a distant stage with a 5-year survival rate (around 3%) 2. Currently, gemcitabine is the prevalent chemotherapeutics strategy for pancreatic cancer treatment 3. However, the lack of miraculous clinical responses and the frequent emerges of chemoresistance prompt the demand to search for more effective chemotherapeutic regimens 4, 5.

As a natural dietary agent, tea (*Camellia sinensis* (L.) O. Kuntze, Theaceae) is increasingly utilized in scientific research and clinical practice by their advantages of high efficacy and low side effects 6. Dark tea is one of the most popular types of Chinese tea, which is mainly produced in Hunan, Yunnan, Hubei, Guangxi and Sichuan provinces. Dark tea is featured by the post-fermented production process 7, which is associated with additional involvement of microorganisms that can result in a visible effect on chemical composition of the tea 8. Given the popularity of dark tea as a beverage in people's daily life, the health benefits from dark tea and the relevant concrete mechanisms attract more and more attention. Previous studies indicate dark tea displays characteristic biological activity in various aspects. Dark tea extract has been found to inhibit lipogenic metabolism by repressing gene expression of sterol regulatory element binding protein-1c and fatty acid synthase and CCAAT/enhancer binding protein α, while promote energy expenditure and lipodieresis through upregulation of gene expressions of hepatic peroxisome proliferator-activated receptor α, carnitine palmitoyltransferase 1a and LDL receptor 9. Dark tea also has been shown to potentially act as the antioxidant and nitric oxide scavenging agent, as exemplified by the finding that dark tea extract exhibits the negative effect on nitric oxide production in lipopolysaccharide-induced RAW 264.7 macrophages 10. Referring to cancer research, a newly identified acylated flavonol glycoside named as Camellikaempferoside A (kaempferol3-O-[E-p-coumaroyl-(→2)][α-l-arabinopyranosyl-(1→3)][α-l-rhamnopyranosyl(1→6)]-β-dglucopyranoside) is isolated from dark tea, which has been shown to exhibit anti-proliferative activity against breast cancer MCF-7 and MDA-MB-231 cells 11.

In this study, we procedurally performed two-rounds of extraction of dark tea, by which water eluate from ethyl acetate extract is identified as the most effective component that can attenuate cell growth of pancreatic cancer. In terms of mechanism, we found water eluate of dark tea leads to an enhancement of p38 activation and concomitant inhibition of p38 produces an addictively negative effect on cell growth of pancreatic cancer. Moreover, cDNA microarray analysis indicates water eluate treatment causes a changed gene expression pattern in pancreatic cancer cells, among which the subsequent analysis demonstrates ID1 is critically involved in cell growth arrest of pancreatic cancer resulted by the dark tea extract.

## Materials and Methods

### Dark tea extract preparation

Three types of dark tea (*Camellia sinensis* (L.) O. Kuntze, Theaceae) named as Fuzhuan tea, Qianliang tea and Tianjian tea, which are used in this study are produced from Anhua County, Yiyang City, Hunan Province. Dark tea leaves (500g) were extracted three times by 10-fold volume boiling water for 2h, 1.5h and 1h, respectively. After combined and concentrated under reduced pressure, the solution was successively extracted with petroleum ether, ethyl acetate, and n-butyl alcohol, and then concentrated under reduced pressure and dried. We ended up with petroleum ether extract, ethyl acetate extract, n-butyl alcohol extract, and the residual water extract. The extracts were dissolved in DMSO and stored at 4℃ until used.

The ethyl acetate extract (26g) from Tianjian dark tea was dissolved in 95% ethyl alcohol and then the solution was combined with HP-20 macroporous resin by mass ratio of 1:2. The compound was loaded into the chromatographic column (3.7cm×24cm) after drying and then performed static adsorption for 24h. Next, the compound was successively eluted with 3-fold column volume of distilled water, of 30% ethyl alcohol, of 50% ethyl alcohol and of 95% ethyl alcohol. After concentrated under reduced pressure and dried, the eluates were dissolved in DMSO and stored at 4℃ until used.

### UHPLC Q-TOF LC/MS analysis and determination of total sugar content

An UHPLC 1290 infinity Ⅱ instrument coupled with a 6554 Quadrupole-Time of Flight (Q-TOF) mass spectrometer (Agilent Technologies, Santa Clara, CA, USA) was employed. Chromatographic separation was achieved on a Zorbax RRHD Eclipse plus C18 column (2. 1 mm ×50 mm, 1. 8 μm) (Agilent Technologies, Santa Clara, CA, USA). Mobile phase (A) was aqueous formic acid (0.1%, v/v), and mobile phase (B) was acetonitrile. A gradient elution program was used: 0 - 2 min, 10% B; 3 - 30 min, 10% - 100% B. The injection volume was 5.0 μL, the flow rate was 0.40 mL.min^-1^ and the column temperature was maintained at 35 ^◦^C. The electrospray source of the MS was operated in positive mode and the MS parameters were: drying gas (N_2_) flow rate, 8.0 L/min; drying gas temperature, 320℃; Sheath gas temperature, 350℃; Sheath gas (N_2_) flow rate, 11.0 L/min; spray voltage, 4000 V; and fragmentor voltage, 175 V. Mass spectra was recorded across the range *m/z* 100-1700 with accurate mass measurement of all mass peaks. The stepped normalized collision energies at auto MS/MS mode were 10, 20, and 40 eV. All the operations, acquisition, and analysis of data were monitored by Agilent LC-Q-TOF-MS MassHunter workstation qualitative Software Version b.08.00 (Agilent Technologies, Santa Clara, CA, USA) and operated under MassHunter workstation Software Version B.09.00 (Agilent Technologies). The databases of TCM_database (Agilent Technologies, Santa Clara, CA, USA) and Metlin_Metabolities_AM_PCDL (Agilent Technologies, Santa Clara, CA, USA) were used for searching potential compounds.

As the dark tea was extracted by hot water, the water-eluate part of macroporous resin might contain some sugar, including small molecules of sugar and polysaccharides. The content of total sugar was determined by the classical phenol - sulfuric acid method. The standard curve was as follows: Y=9.2593X+0.0826 (R^2^=0.9994), Where Y represents the concentration (mg/mL) of sugar, and X represents the UV absorbance at 490 nm. Standard curve concentration ranges were from 0.004mg/mL to 0.024mg/mL. As a result, the total sugar content of the water-eluate part of macroporous resin was 5.67%.

### Cells culture

The SW1990, PANC-1, SW1116 and HPDE cells were maintained in Dulbecco's modified Eagle's medium (DMEM) supplemented with 10% fetal bovine serum (FBS), 100 μ units/ml streptomycin and incubated at 37℃ in a humidified atmosphere containing 5% CO2 in air. All the cell lines were obtained from the American Type Culture Collection (ATCC) and routinely tested for mycoplasma contamination. The cell lines were not authenticated.

### Cell proliferation and cytotoxicity assay

The effect of the dark tea extract on proliferation of SW1990, PANC-1, SW1116 and HPDE cells was evaluated by the cell counting kit-8 (CCK8, Dojindo) assay. SW1990 or PANC-1, SW1116 and HPDE were plated at 3×10^3^ or 5×10^3^ density in a 100-µL volume in 96-well plates, cells were treated for 48h with various concentration of extract or eluate. At the end of the treatment intervals, 10 µL of CCK8 solution was added into each well. After 2h of incubation in a 37℃ and 5% CO_2_ incubator, the absorbance was measured in a microplate reader at a wavelength of 450 nm.

### Immunoblotting analysis

Cells were lysed with RIPA lysis buffer and Phenylmethyl sulfonyl fluoride (PMSF, A610425, Sangon Biotech). The protein concentration was determined by a BCA protein assay kit (Thermo). Equal amount of protein samples in SDS sample buffer (1% SDS, 62.5 mM Tris-HCl, pH 6.8, 10% glycerol, 5% mercaptoethanol, and 0.05% bromphenol blue) were boiled for 5min and subjected to reducing 10% SDS-PAGE. Proteins were separated by SDS-PAGE and electrophoretically transferred to PVDF membrane. The membrane was blocked with 5% non-fat milk powder in PBST at room temperature for 2h, and then incubated with primary antibodies at 4℃ overnight. Following washing, the membrane was incubated with secondary antibody conjugated with horseradish peroxidase (anti-rabbit or anti-mouse) at room temperature. The following antibodies were used: antibodies against phosphor-MAPKAPK-2 (Thr334) (#3041, 1:1000), and AKT (9272S, 1:1000) were purchased from Cell Signaling Technology; antibodies against AKT (phospho-ser473) (11054, 1:1000), ERK1/2 (phospho-Thr202) (12082, 1:1000) and ERK1/2 (29162, 1:1000) were purchased from Signalway Antibody; antibody against V5-tag (66007-1-Ig, 1:1000) was purchased from Proteintech.

### ROS (reactive oxygen species) detection assay

Cells were seeded onto 12-well plates and treated with half maximal inhibitory concentration (IC50) of extract or eluate in various length of time in the absence or presence of a p38 inhibitor, SB203580 (S1076, Selleck). After completion of treatment time, cells were washed once with PBS to avoid the possible interference with the experiment. Thereafter, cells were incubated with 5 μM H2DCFDA (HY-D0940, MCE) in fresh medium in the dark for 30 min at 37℃, then harvested with 0.25% trypsin-EDTA solution (gibco) and washed once with ice-cold PBS. Finally, cells were suspended in PBS and immediately analyzed with flow cytometer (BD Biosciences AccuriC6, USA).

### cDNA microarray analysis

IC50 of water eluate from ethyl acetate extract of Tianjian dark tea was used to treat SW1990 cells for 12h. Total RNA was isolated using RNeasy mini kit (Qiagen, Germany). Pairedend libraries were synthesized by using the TruSeq™ RNA Sample Preparation Kit (Illumina, USA) following TruSeq™ RNA Sample Preparation Guide. Briefly, the poly-A containing mRNA molecules were purified using poly-T oligo-attached magnetic beads. Following purification, the mRNA is fragmented into small pieces using divalent cations under 94℃ for 8 min. The cleaved RNA fragments are copied into first strand cDNA using reverse transcriptase and random primers. This is followed by second strand cDNA synthesis using DNA Polymerase I and RNase H. These cDNA fragments then go through an end repair process, the addition of a single 'A' base, and then ligation of the adapters. The products are then purified and enriched with PCR to create the final cDNA library. Purified libraries were quantified by Qubit® 2.0 Fluorometer (Life Technologies, USA) and validated by Agilent 2100 bioanalyzer (Agilent Technologies, USA) to confirm the insert size and calculate the mole concentration. Cluster was generated by cBot with the library diluted to 10 pM and then was sequenced on the Illumina HiSeq 2500 (Illumina, USA). The library construction and sequencing were performed at Shanghai Biotechnology Corporation.

### RNA isolation, reverse transcription and real-time PCR

IC50 of water eluate from ethyl acetate extract of Tianjian dark tea was used to treat cells for 12h. Total RNA was isolated from cells by using Trizol reagent (Ambion) according to the manufacturer's protocol. Complementary DNA was produced from cellular RNA (1 μg) using PrimeScript^TM^ RT reagent kit with gDNA Eraser (RR047A, TaKaRa). Reverse-transcribed cDNA in triplicate samples was checked for target mRNA level by quantitative real-time PCR with Power SYBR Green PCR Master Mix (Thermo). The qPCR primer sequences were: hACTB: 5'- ACAATGTGGCCGAGGACTTTGA-3' (forward) and 5'- TGTGTGGACTTGGGAGAGGACT-3' (reverse); hID1: 5'- CTACGACATGAACGGCTGTTA-3' (forward) and 5'- CAACTGAAGGTCCCTGATGTAG-3' (reverse); hPYCARD: 5'- CTCAAGAAGTTCAAGCTGAAGC-3' (forward) and 5'- TAGGTCTCCAGGTAGAAGCTG-3' (reverse).

### Transfection assay

SW1990 cells were transfected with various plasmids using Lipofectamine 2000 (Invitrogen) according to the vendor's instructions. The pLX304 human ID1 was generated with the oligonucleotides 5'-CGCAAATGGGCGGTAGGCGTG-3' and 5'-TACGGGAAGCAATAGCATGA-3'. The pLX304 controls were generated with control oligonucleotides 5'-CGCAAATGGGCGGTAGGCGTG-3' and 5'-CATAGCGTAAAAGGAGCAACA-3'.

### Experiment of xenograft mouse tumor model

All animal experiments conformed to the guidelines of the Institutional Animal Care and Use Committee of Shanghai Jiaotong University. Mice were maintained in a temperature- and light-controlled environment with *ad libitum* access to water. The mice were randomly put into separate groups/cages for experiments and received a standard chow diet. Five-week-old male nu/nu mice were subcutaneously injected with 1 × 10^7^ PANC-1 cells in a volume of 200µl of PBS. Two weeks after cell inoculation, the mice were randomly divided into two groups (*n*=6 per group). One group received intraperitoneal injection of dark tea extract (300 mg/kg/day) for 21 days as compared with the other group injected with DMSO (control group). Tumor volume was measured by using length *(a)* and width *(b)* and calculated using the equation: *V=ab^2^/2* with vernier calipers.

## Results

### Ethyl acetate extract from dark tea inhibits growth of pancreatic cancer cells

**Figure 1:**
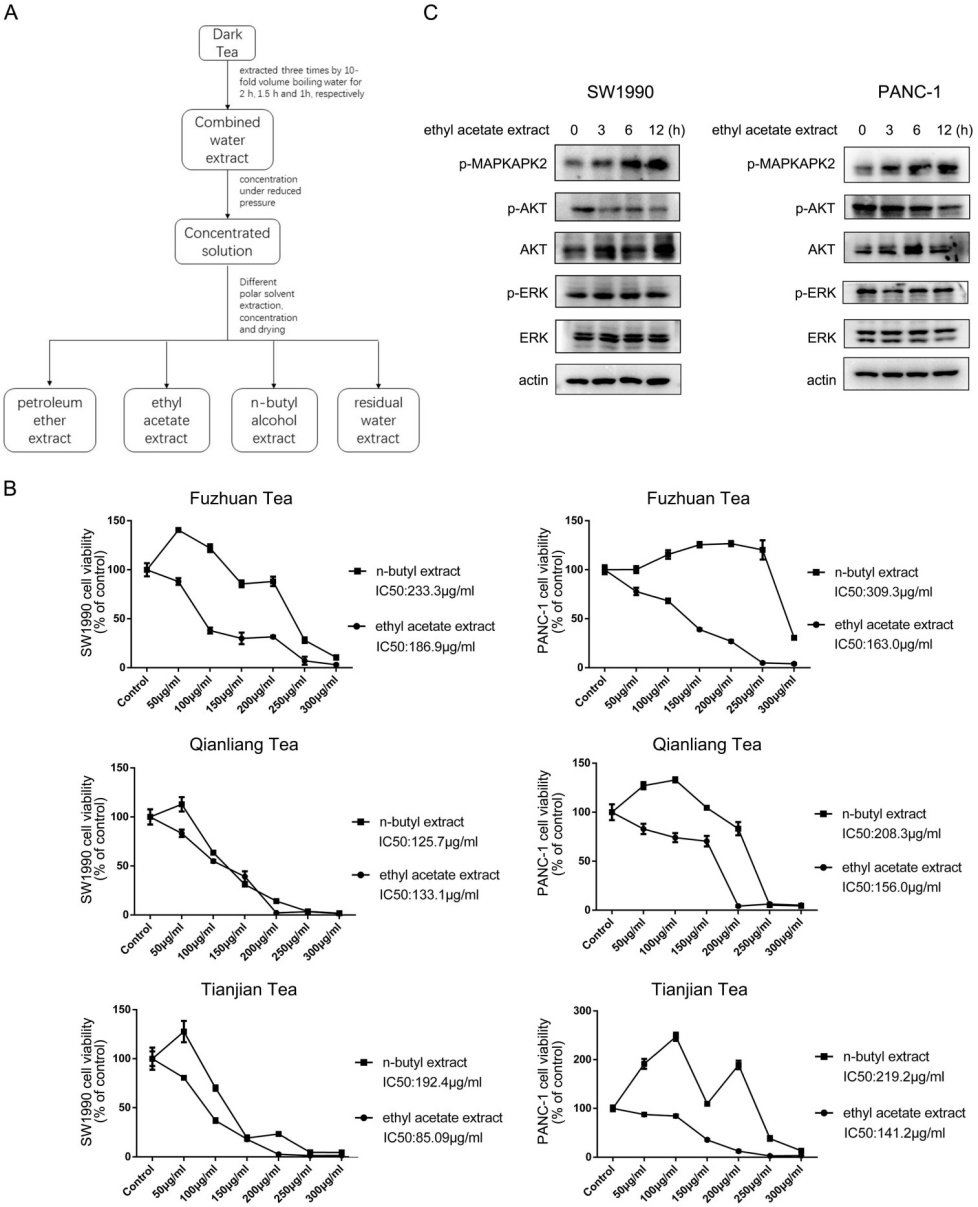
** Ethyl acetate extract from dark tea inhibits growth of pancreatic cancer cells.** (A) The indicated dark tea was extracted three times by 10-fold volume boiling water for 2 h, 1.5 h and 1h, respectively. After combined and concentrated under reduced pressure, the solution was successively extracted with petroleum ether, ethyl acetate, and n-butyl alcohol, and then concentrated under reduced pressure and dried. (B) Various concentration of ethyl acetate extract or n-butyl alcohol extract from indicated types of dark tea were used to treat PANC-1 or SW1990 cells for 48 h. CCK8 assay was performed. The values are presented as mean ± s.e.m. (n=3 independent experiments). (C) IC50 (Half maximal inhibitory concentration) for cell growth of ethyl acetate extract of Tianjian dark tea was used to treat SW1990 and PANC-1 cells for indicated length of time. Immunoblotting analysis was performed using indicated antibodies.

To analyze the potential impact of dark tea (*Camellia sinensis* (L.) O. Kuntze, Theaceae) extract on pancreatic cancer cells growth, three types of dark tea named as Fuzhuan tea, Qianliang tea and Tianjian tea, which are produced from Anhua County, Yiyang City, Hunan Province, were chosen for the investigation. Tea extracts were isolated according to solvent extraction method, ended up with petroleum ether extract, ethyl acetate extract, n-butyl alcohol extract, and the residual water extract (Figure 1A), and ethyl acetate extract and n-butyl alcohol extract were selected to test their potential effect on the growth of pancreatic cancer cells. As shown in Figure 1B, CCK8 analyses indicated ethyl acetate extract from all three types of dark tea exerted a more profound inhibitory effect on cell growth than n-butyl alcohol extract in SW1990 and PANC-1 human pancreatic cancer cells, which was shown in a dosage dependent manner. The relevant calculation indicated ethyl acetate extract from Tianjian tea has smaller IC50 (Half maximal inhibitory concentration) for cell growth than that of other two types of tea, which are 85.09 μg/ml and 141.2μg/ml in SW1990 and PANC-1 cells respectively (Figure 1B). Based on this, ethyl acetate extract from Tianjian tea was further utilized to perform subsequent analysis. Time course of immunoblotting analysis indicated addition of ethyl acetate extract apparently increased phosphorylation of MAPKAPK2, a downstream substrate of p38 12, both in SW1990 and PANC-1 cells (Figure 1C). At the meantime, AKT but not ERK phosphorylation was found to be significantly decreased by ethyl acetate extract treatment in both cell lines (Figure 1C). Given the well-known implication of p38 and AKT signaling in cell growth 13, 14, these results suggest ethyl acetate extract of dark tea impedes cancer cells growth via its effects on p38 and AKT activity.

### Water eluate from ethyl acetate extract is the effective component to inhibit cell growth

**Figure 2:**
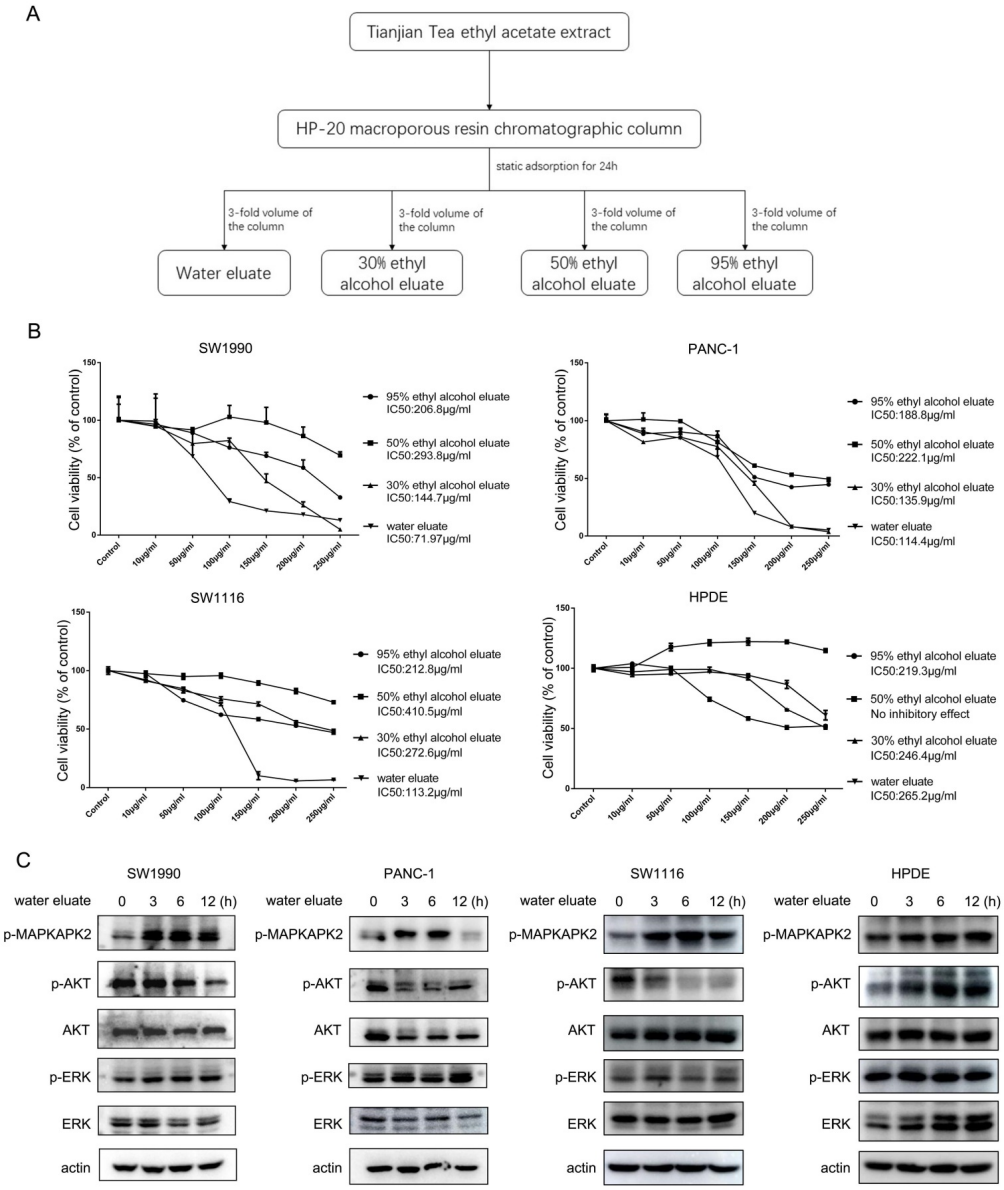
** Water eluate from ethyl acetate extract is the effective component to inhibit cell growth.** (A) The ethyl acetate extract from Tianjian dark tea was dissolved in 95% ethyl alcohol and then the solution was combined with HP-20 macroporous resin by mass ratio of 1:2. The compound was loaded into the chromatographic column after drying and then performed static adsorption for 24h. Next, the compound was successively eluted with distilled water, 30% ethyl alcohol, 50% ethyl alcohol, 95% ethyl alcohol. (B) The indicated concentration of eluate from ethyl acetate extract of Tianjian dark tea were used to treat SW1990, PANC-1, SW1116 or HPDE cells for 48h. CCK8 assay was performed. The values are presented as mean ± s.e.m. (n=3 independent experiments). (C) IC50 of water eluate from ethyl acetate extract of Tianjian dark tea was used to treat SW1990, PANC-1, SW1116 or HPDE cells for indicated length of time. Immunoblotting analysis was performed using indicated antibodies.

Ethyl acetate extract was further fractioned into components of water eluate, 30% ethyl alcohol eluate, 50% ethyl alcohol eluate and 95% ethyl alcohol eluate (Figure 2A) and their impact on cell growth was examined. Interestingly, we found the extract of water eluate was the most efficient component to restrain the growth of SW1990 and PANC-1 cells, in which IC50 values were 71.97 μg/ml and 114.4 μg/ml respectively (Figure 2B, upper panels). Meanwhile, the inhibitory effect of water eluate was also observed in SW1116 human colorectal cancer cells, in which the IC50 value was 113.2 μg/ml (Figure 2B, left-bottom panel). In contrast, water eluate displayed a much lower inhibitory efficiency for cell growth in HPDE human normal pancreatic duct epithelial cells (IC50 value: 265.2 μg/ml) (Figure 2B, right-bottom panel). In accordance with results shown above, addition of water eluate apparently resulted in increased MAPKAPK2 phosphorylation and the concomitant dropped AKT phosphorylation in SW1990, PANC-1 and SW1116 cells but not in HPDE cells (Figure 2C). These data reveal water eluate is the primary fraction responsible for the effects of ethyl acetate extract on cell growth and responsive signaling in cancer cells.

### The constituents and content of total sugar of water eluate from ethyl acetate extract are examined

**Figure 3:**
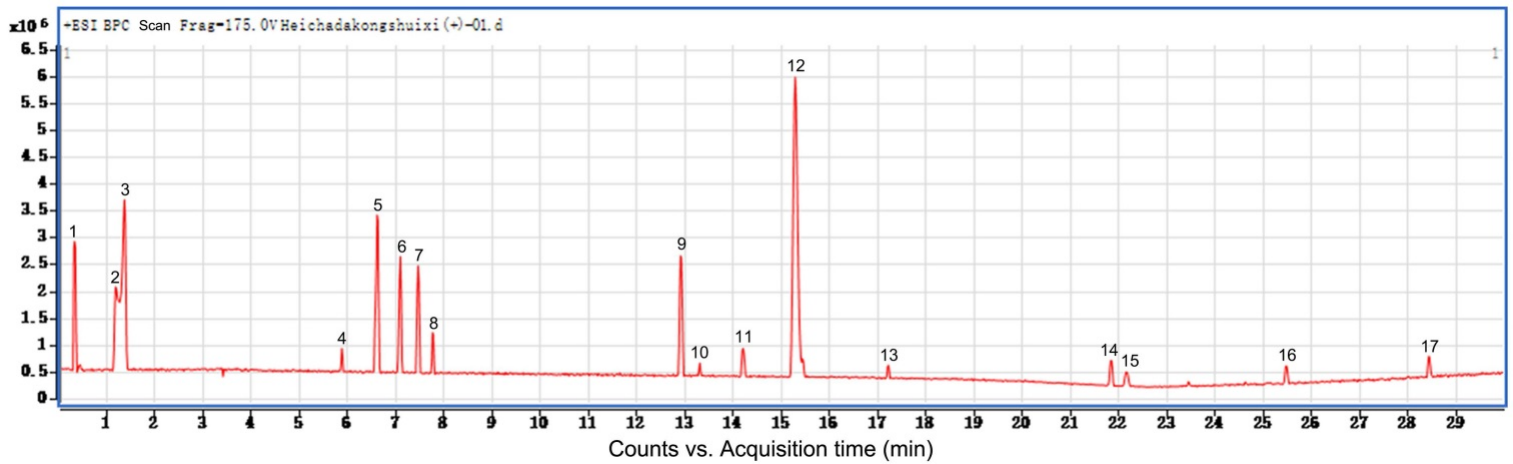
** The constituents of water eluate from ethyl acetate extract are examined.** LC/MS method was employed to analyze the primary chemical profile of water eluate from ethyl acetate extract. All the operations, acquisition, and analysis of data were monitored by Agilent LC-Q-TOF-MS MassHunter workstation qualitative Software Version B.08.00 and operated under MassHunter workstation Software Version B.09.00.

Additionally, we performed preliminary component analysis of water eluate from ethyl acetate extract by LC/MS analysis (Figure 3), and seventeen constituents mainly including catechins, flavonoids and organic acids were tentatively identified based on the information about chemistry of dark tea in literature and by searching databases of Agilent TCM_database and Metlin_Metabolities_AM_PCDL (Table 1). Meanwhile, the content of total sugar was determined by the classical phenol-sulfuric acid method, which shows the total sugar content of the water eluate part of macroporous resin was 5.67% (Table 2).

### Water eluate has a synergic inhibitory effect with p38 inhibition on cancer cells growth

**Figure 4:**
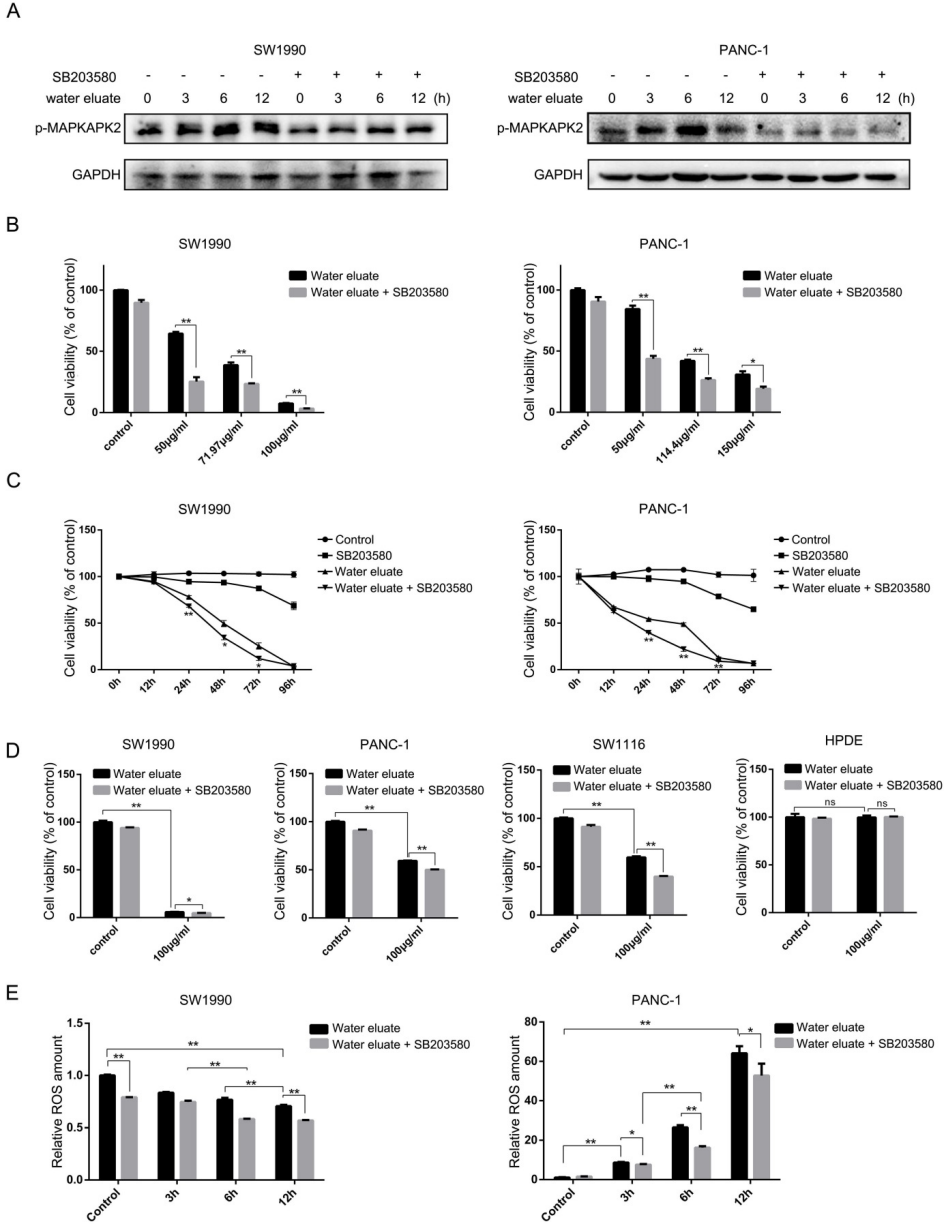
**Water eluate has a synergic inhibitory effect with p38 inhibition on cancer cells growth.** (A) SW1990 or PANC-1 cells pretreated with or without SB203580 (10μM for 1h), were treated with IC50 of water eluate from ethyl acetate extract of Tianjian dark tea for indicated length of time. Immunoblotting analysis was performed using indicated antibodies. (B) SW1990 or PANC-1 cells pretreated with or without SB203580 (10μM for 1h) were treated with indicated dosage of water eluate from ethyl acetate extract of Tianjian dark tea for 48 h. CCK8 assay was performed. (C) SW1990 or PANC-1 cells pretreated with or without SB203580 (10μM for 1h), were treated with IC50 of water eluate from ethyl acetate extract of Tianjian dark tea for indicated length of time. CCK8 assay was performed. (D) SW1990, PANC-1, SW1116 or HPDE cells pretreated with or without SB203580 (10μM for 1h), were treated with water eluate from ethyl acetate extract of Tianjian dark tea (100μg/ml) for 48 h. CCK8 assay was performed. (E) SW1990 or PANC-1 cells pretreated with or without SB203580 (10μM for 1h), were treated with IC50 of water eluate from ethyl acetate extract of Tianjian dark tea for indicated length of time. ROS amount was measured. In B-E, the values are presented as mean ± s.e.m. (n=3 independent experiments), * represents P <0.05, ** represents P <0.01, ns represents no significant (Student's t-test) between the indicated groups.

We wondered that whether p38, whose activity was increased by tea extract treatment (Figure 1C and Figure 2C), would act as a critical effector that mediates tea extract-induced cellular events. To this end, water eluate was used to treat SW1990 or PANC-1 cells in various length of time in the absence or presence of SB203580, a p38 inhibitor, of which the inhibitory activity was validated by its effect on MAPKAPK2 phosphorylation (Figure 4A). Functional analysis indicated water eluate inhibited cell growth of SW1990 and PANC-1 cells in a dosage dependent manner, which was unexpectedly exacerbated by SB203580 treatment (Figure 4B). In addition, time course analysis further revealed the cooperative suppressive-effect of water eluate and SB203580 treatment on SW1990 and PANC-1 cells growth (Figure 4C). These data imply the enhanced p38 signaling resulted by tea extract would be a cellular feedback to sustain cancer cells growth. Consistent with the observations in SW1990 and PANC-1 cells (Figure 4D, the 1^st^ and the 2^nd^ panels), treatment of water eluate (100μg/ml) notably suppressed SW1116 cells growth and co-treatment with SB203580 enhanced the inhibitory effect in a significant level (Figure 4D, the 3^rd^ panel). In contrast, neither water eluate treatment alone nor simultaneous treatment with water eluate and SB203580 resulted in a significant effect on HPDE cells growth (Figure 4D, the 4^th^ panel). Furthermore, the potential effect of water eluate and SB203580 co-treatment on intracellular ROS production was examined in SW1990 and PANC-1 cells. As a result, water eluate addition decreased intracellular ROS accumulation in SW1990 cells, and this effect was further enhanced by SB203580 treatment to certain degree (Figure 4E, left panel). On the contrary, we found water eluate addition led to an increased accumulation of intracellular ROS in PANC-1 cells, which was partially attenuated by SB203580 treatment (Figure 4E, right panel)*.* These inconsistent results suggest the synergic suppressive effect of water eluate with p38 inhibition on cancer cells growth would not be closely associated with the alteration of ROS accumulation.

### Water eluate leads to a changed gene expression profile linking to cell growth arrest

**Figure 5:**
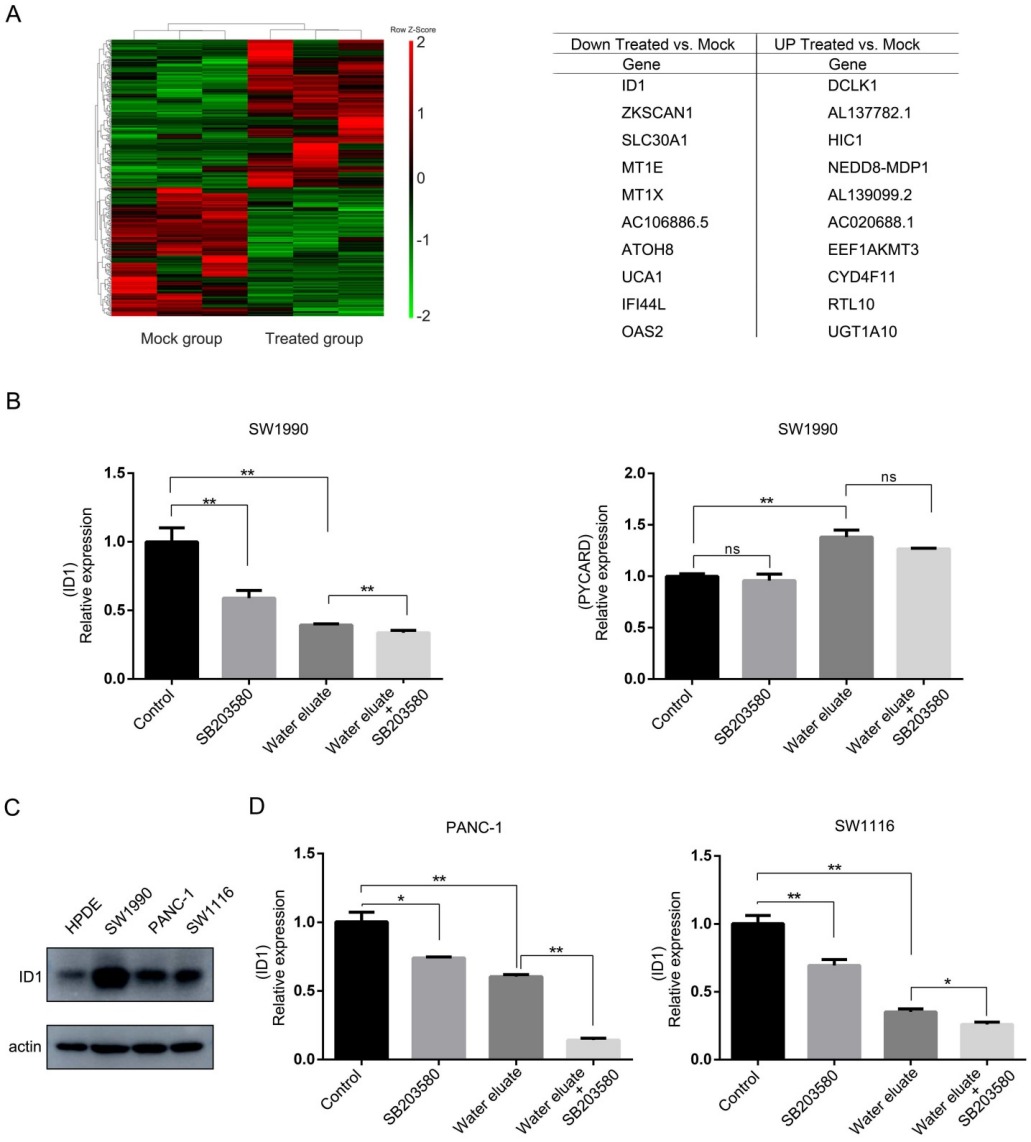
** Water eluate leads to a changed gene expression profile linking to cell growth arrest.** (A) IC50 of water eluate from ethyl acetate extract of Tianjian dark tea was used to treated SW1990 cells for 12 h. cDNA microarray analysis was performed. Hierarchical clustering of 27496 probe sets correlating with water eluate-treated cells show those genes in separating cases from Mock group of cells (left panel). Genes with the largest changes in treated-cells to Mock group (right panel). (B) SW1990 cells pretreated with or without SB203580 (10μM for 1h), were treated with IC50 of water eluate from ethyl acetate extract of Tianjian dark tea for 12 h. Relative mRNA levels of *ID1* (left panel) and *PYCARD* (right panel) were analyzed by real-time PCR. (C) The protein level of ID1 was analyzed by immunoblotting analysis using indicated antibodies. (D) PANC-1 or SW1116 cells pretreated with or without SB203580 (10μM for 1h), were treated with IC50 of water eluate from ethyl acetate extract of Tianjian dark tea for 12 h. Relative mRNA levels of *ID1* were analyzed by real-time PCR. In B and D, the values are presented as mean ± s.e.m. (n=3 independent experiments), * represents P <0.05, ** represents P <0.01, ns represents no significant (Student's t-test) between the indicated groups.

We next examined the effect of water eluate on the global gene expression profile in SW1990 cells through cDNA microarray analysis. Notably, a number of transcripts with the changed level were enriched for genes implicated in anti-proliferation, and apoptosis (Figure 5A), in which *ID1* gene and *PYCARD* gene, whose function are known to be related to cell growth 15-17, were selected to evaluate the physiological impact from water eluate. In line with results from cDNA microarray analysis, qPCR analysis indicated water eluate treatment led to impaired gene transcription of *ID1* while increased gene transcription of *PYCARD* in SW1990 cells (Figure 5B). Additionally, we found *ID1* expression was further decreased by additional SB203580 treatment in SW1990 cells (Figure 5B, left panel). However, no significant addictive effect from co-treatment of water eluate with SB203580 was observed on *PYCARD* expression in SW1990 cells (Figure 5B, right panel). Immunoblotting analysis indicated the protein level of ID1 was notably higher in SW1990, PANC-1 and SW1116 cells than in HPDE cells (Figure 5C). Similar to the results in SW1990 cells, water eluate largely inhibited *ID1* transcription and the synergistic effect from water eluate and SB203580 was observed in PANC-1 and SW1116 cells (Figure 5D). These data suggest ID1 would be importantly involved in the cooperative effect of water eluate and p38 inhibition on cancer cells growth.

### ID1 overexpression exerts rescue effects on cell growth of water eluate-treated tumor cells

**Figure 6:**
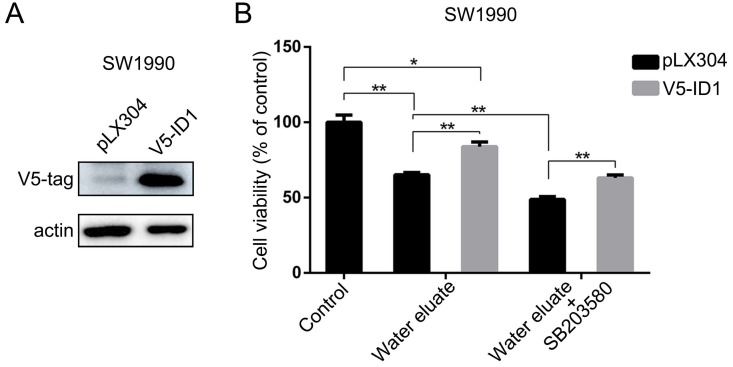
** ID1 overexpression exerts rescue effects on cell growth of water eluate-treated tumor cells.** (A) SW1990 cells were transfected with the plasmid for overexpressing ID1. Immunoblotting analysis was performed using indicated antibodies. (B) SW1990 cells overexpressed with or without ID1 were treated with IC50 of water eluate from ethyl acetate extract of Tianjian dark tea for 48 h in presence or absence of SB203580 (10μM) pretreatment for 1h. CCK8 assay was performed. The values are presented as mean ± s.e.m. (n=3 independent experiments), * represents P <0.05, ** represents P <0.01 (Student's t-test) between the indicated groups.

In light of the results shown above, we set forth to further investigate the physiological role of ID1 on the cell growth of pancreatic cancer cells under tea extract treatment. To this end, ID1 was stably expressed in SW1990 cells (Figure 6A). As shown in Figure 6B, overexpression of ID1 evidently ameliorated cell viability of SW1990 cells either at the condition of water eluate treatment alone or simultaneous treatment of water eluate and SB203580. These results demonstrate ID1 is the critical downstream responsive effector that promotes cell growth arrest of pancreatic cancer cells under tea extract treatment with p38 inhibition.

### Water eluate inhibits cancer cells growth in xenograft mice tumor model

**Figure 7:**
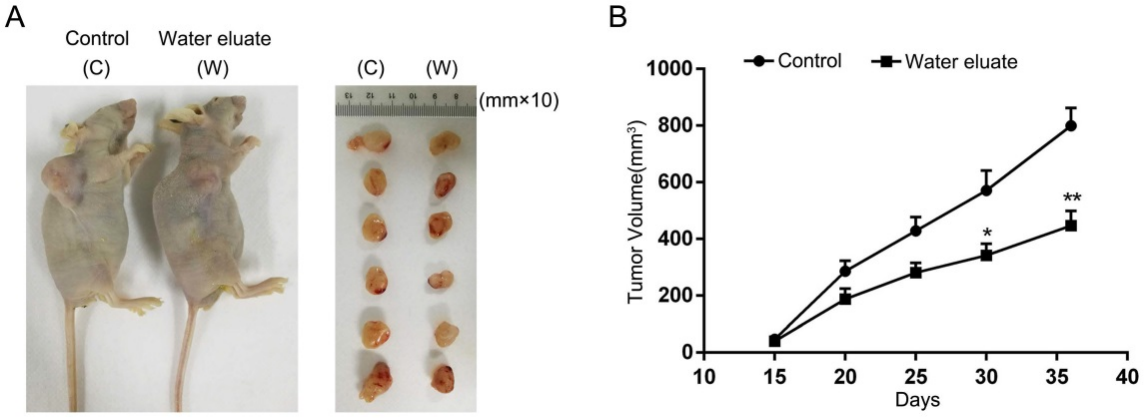
** Water eluate inhibits cancer cells growth in xenograft mice tumor model.** (A) A total of 1 × 10^7^ PANC-1 cells were subcutaneously injected into the athymic nude mice. Water eluate from ethyl acetate extract of Tianjian dark tea (300mg/kg/day) was injected intraperitoneally for 21 days as compared with the other group injected with DMSO (control group). Representative tumor xenografts were shown. (B) Tumor volumes were measured by using length *(a)* and width *(b)* and calculated using the following equation: *V = ab^2^/2*. The values are presented as mean ± s.e.m. (n=6 per group), * represents P <0.05, ** represents P <0.01 (Student's t-test) between the indicated groups.

To further assess the physiological effect of water eluate on tumorigenesis, PANC-1 cells were subcutaneously injected into athymic nude mice, and water eluate or DMSO (control group) was injected intraperitoneally post two weeks. As a result, tumor cells of control group elicited rapid tumor growth (Figure 7A). In contrast, water eluate treatment exerted a repressive effect on tumorigenesis (Figure 7A), in which the prominent difference in tumor growth was observed between water eluate-treatment group and control group after intraperitoneal injection for 21 days (Figure 7B). Altogether, these data reveal the inhibitory effect of water eluate extract on pancreatic tumor growth *in vivo*.

## Discussion

As one of the most popularly consumed Chinese tea (*Camellia sinensis* (L.) O. Kuntze, Theaceae), dark tea causes more and more attention in terms of its physiological implication in various aspects including human metabolism and cancer. For instance, Camellikaempferoside A, a component isolated from dark tea, is reported to inhibit proliferation of breast cancer cells 11. More comprehensive studies are required for deeply understanding the functional effect of dark tea on tumorigenesis. In present study, we first show that ethyl acetate extract of dark tea exhibits a significantly repressive effect on cell growth in pancreatic cancer cells, which is accompanied by enhanced MAPKAPK2 phosphorylation and decreased AKT phosphorylation. Subsequently, the second round of extraction based on ethyl acetate extract is performed and we found the fraction of water eluate is the most effective fraction that impairs cancer cells growth and tumor development *in vivo*.

Consistently, treatment of water eluate leads to a similar effect to ethyl acetate extract on status of MAPKAPK2 and AKT phosphorylation. Intriguingly, further functional study indicates p38 inhibition does not ameliorate but potentiates the negative effect of water eluate on cell growth of pancreatic cancer. Furthermore, ectopic expression of ID1, whose expression we noted is decreased under water eluate treatment, evidently attenuates the negative impact of water eluate on cell growth. Taken together, this study fractionizes dark tea according to the functional relevance to cancer cells growth, and demonstrates that ID1 would be a pivotal downstream effector that mediates the cell growth-inhibitory effects from water eluate fraction. Of note, the lower inhibitory efficiency of dark tea extract for cell growth in HPDE cells than in cancer cells is observed in our study (Figure 2B), which would be related to the limited ID1 expression levels in HPDE cells (Figure 5C), as ID1 levels was found to be positively related to cancer cells growth (Figure 6B). This suggests the component of water eluate fraction could be potentially developed into an effective tumor-suppressing substance.

p38 activity has been implicated in the regulation of cell proliferation, differentiation and survival in various cancer cell lines 18. Distinct studies indicated p38 can play as either the tumor suppressor or promoter roles, which would be dependent on the stage of tumor development and the relevant genetic context 19, 20. In our study, the fraction of water eluate results in p38 activation as indicated by MAPKAPK2 phosphorylation, and functional analysis shows concomitant p38 inhibition exacerbates the suppressive effects of water eluate on cancer cells growth. These observations suggest increased p38 activity under water eluate treatment would be a protective feedback signal that maintains sustainable cancer cells growth. In other words, p38 activity here circumstantially conducts a positive effect on cancer cells growth under water eluate treatment. This point is further supported by the data that p38 inhibition and water eluate produce a synergic inhibitory effect on the expression of ID1, ectopic expression of which we found significantly reversed cancer cells growth. In consistence, previous studies have shown that *ID1* mRNA species are highly expressed in pancreatic cancer samples by comparison with normal or chronic pancreatitis samples, whose depletion prevents the proliferation of pancreatic cancer cells 15, 21. Of note, our microarray analysis identified multiple genes whose expressions are responsive to water eluate treatment. In this regard, the potential impact of these genes on the growth of pancreatic cancer cells is worthy of further investigation. Altogether, our findings provide a theoretical basis that would facilitate for further study against functional separation of dark tea in future, which could be beneficial for uncovering the unknown relevant physiological importance of concrete components.

## Conclusion

In brief, this study demonstrated that water eluate fraction from ethyl acetate extract of dark tea (*Camellia sinensis* (L.) O. Kuntze, Theaceae) was able to suppress cell growth and tumorigenesis in pancreatic cancer cells. Further, simultaneous treatment of water eluate fraction and p38 inhibitor resulted in a synergic inhibitory effect on pancreatic cancer cell growth. Moreover, the transcription of *ID1* was found to be repressed by water eluate fraction, and its downregulation was required for dark tea extract or/and p38 inhibition-suppressed growth of pancreatic cancer cells. Hence, our data revealed the potential of a specific fraction of dark tea as an alternative agent for clinical treatment in pancreatic cancer therapy and the relevant molecular mechanism.

## Figures and Tables

**Table 1:** LC-MS/MS analysis of water eluate from ethyl acetate extract from Tianjian dark tea

1	Epigallocatechin gallate
2	Caprylic acid
3	Catechin or Epicatechin
4	Isoscutellarein
5	Quercetin-3-*α*-rhamnosyl(1→4)-α-rhamnosyl(1→6)-*β*-glucoside
6	Cerebroside Ⅰ
7	Cistanoside A
8	Platycogenica acid C
9	Shanzhiside
10	Methylpalmitate
11	Menthyl acetate
12	Harpagoside
13	Suavedic acid
14	Erectquinone B
15	Salviol
16	5-β-cholanic acid
17	Merulinic acid A

**Table 2:** Phenol-sulfuric acid analysis of total sugar content of water eluate from ethyl acetate extract from Tianjian dark tea

Standard curve	Concentration range	Total sugar content
Y=9.2593X+0.0826 (R^2^=0.9994)	0.004mg/mL-0.024mg/mL	5.67%

## Abbreviations

LDL: Low density lipoprotein
ID1: Inhibitor of differentiation protein 1
DMSO: Dimethyl sulfoxide
LC/MS: Liquid chromatography-mass spectrometry
CCK8: Cell counting kit-8
SDS-PAGE: Sodium dodecyl sulfate-polyacrylamide gel electrophoresis
PVDF: Polyvinylidene fluoride
IC50: Half maximal inhibitory concentration
ROS: Reactive oxygen species
PYCARD: Apoptosis-associated speck-like protein containing a CARD
qPCR: Quantitative real-time PCR
